# Validity and sensitivity of field tests’ heart-rate recovery assessment in recreational football players

**DOI:** 10.1371/journal.pone.0282058

**Published:** 2023-03-01

**Authors:** Susana Póvoas, Peter Krustrup, Carlo Castagna

**Affiliations:** 1 Research Center in Sports Sciences, Health Sciences and Human Development, CIDESD, University of Maia, ISMAI, Maia, Portugal; 2 Department of Sports Science and Clinical Biomechanics, SDU Sport and Health Sciences Cluster (SHSC), University of Southern Denmark, Odense, Denmark; 3 Department of Physical Education and Sports Training, Shanghai University of Sport, Shanghai, China; 4 Sport and Health Sciences, College of Life and Environmental Sciences, University of Exeter, Exeter, United Kingdom; 5 Fitness Training and Biomechanics Laboratory, Italian Football Federation (FIGC), Technical Department, Coverciano (Florence), Italy; 6 Department of Biomolecular Sciences, School of Exercise and Health Sciences, Carlo Bo Urbino University, Urbino, Italy; Universidade Federal de Mato Grosso do Sul, BRAZIL

## Abstract

We aimed at examining the criterion validity and sensitivity of heart-rate recovery (HR_Rec_) in profiling cardiorespiratory fitness in male recreational football players in the untrained and trained status, using endurance field-tests. Thirty-two male untrained subjects (age 40 ± 6 years, VO_2max_ 41.7 ± 5.7 ml·kg^-1^·min^-1^, body mass 82.7 ± 9.8 kg, stature 173.3 ± 7.4 cm) participated in a 12-week (2‒3 sessions per week) recreational football intervention and were tested pre- and post-intervention (i.e. untrained and trained status). The participants performed three intermittent field tests for aerobic performance assessment, namely Yo-Yo intermittent endurance level 1 (YYIE1) and level 2 (YYIE2) tests, and Yo-Yo intermittent recovery level 1 (YYIR1) test. VO_2max_ was assessed by performing a progressive maximal treadmill test (TT) and maximal HR (HR_max_) determined as the maximal value across the testing conditions (i.e., Yo-Yo intermittent tests or TT). HR_Rec_ was calculated as the difference between Yo-Yo tests’ HR_peak_ or HR_max_ and HR at 30 s (HR_30_), 60 s (HR_60_) and 120 s (HR_120_) and considered as beats·min^-1^ (absolute) and as % of tests’ HR_peak_ or HR_max_ values. Significant post-intervention improvements (p<0.0001) were shown in VO_2max_ (8.6%) and Yo-Yo tests performance (23–35%). Trivial to small (p>0.05) associations were found between VO_2max_ and HR_Rec_ (r = -0.05−0.27, p>0.05) across the Yo-Yo tests, and training status either expressed as percentage of HR_peak_ or HR_max_. The results of this study do not support the use of field-test derived HR_Rec_ to track cardiorespiratory fitness and training status in adult male recreational football players.

## Introduction

Heart rate (HR) monitoring is a valid and popular method for controlling aerobic training aimed at enhancing cardiorespiratory health [1]. In individuals with different training status, health conditions, age and sex, maximal exercise and recovery HR are the variables usually considered to prescribe and monitor training, and to assess cardiorespiratory fitness [1, 2]. Heart rate recovery (HR_Rec_) is most commonly measured as the rate at which heart rate decreases within the following seconds/minutes after the end of exercise [3, 4] and reflects the dynamic balance and coordinated interplay between parasympathetic reactivation and sympathetic withdrawal [4–6].

HR_Rec_ following exercise to exhaustion was deemed sensitive to the interplay between parasympathetic and sympathetic nervous activity, reflecting autonomic efficiency [2, 3, 7]. This, alongside with the high accessibility to HR_Rec_ measurements, promoted the development of normative values considered useful for detecting pernicious variations in post-maximal-exercise HR kinetics in daily practice [3, 8–10]. Indeed, faster HR_Rec_ was reported to be associated with a higher fitness level, and subjects with abnormal HR_Rec_ (i.e. a decrease of ≤ 12 beats·min^-1^ for HR at 60 s after the end of the test) [3, 8] were less likely to be engaged in regular and strenuous exercise [8]. Furthermore, HR_Rec_ revealed to be a prognostic indicator of adverse cardiometabolic outcomes and an independent factor for metabolic syndrome prediction [11, 12]. The published scientific evidence of a deleterious effect of attenuated HR_Rec_ on cardiovascular and metabolic health and all-cause mortality, promoted HR_Rec_ recording in clinical practice as “per se” routine for health risk assessment [12].

Training interventions using conventional aerobic exercise promoted positive changes in HR_Rec_ in cardiovascular patients and in athletes [13, 14]. However, most of the published research studies were carried out considering pre- to post-intervention HR_Rec_ at selected time points post-exercise, as training outcome variables, not considering changes in VO_2max_. In fact, the supposed low sensitivity of VO_2max_ for tracking cardiorespiratory fitness changes, and its low absolute reliability, promoted the consideration of HR_Rec_ for performance monitoring [13]. Nevertheless, VO_2max_ levels relate to cardiorespiratory health and to the likelihood of all-cause or cardiovascular mortality, suggesting consideration of VO_2max_ tracking in cardiorespiratory fitness enhancing programmes [15]. The practical interest in evaluating cardiorespiratory fitness with an easily accessible variable like HR_Rec_ and with endurance field tests, warrants therefore experimental consideration in recreational sports [16, 17].

Recreational football research has provided compelling evidence of clinically sound training-induced improvements in cardiorespiratory fitness and aerobic performance [18, 19]. Regular weekly practice of recreational football in the form of small-sided games, has been proposed as an alternative exercise mode for improving cardiovascular health across age, sex and health status. The casually intermittent nature of recreational football and the associated variability of the individual responses to practice (i.e. small-sided games), suggests the periodical evaluation of aerobic fitness to assess the effectiveness of the training programmes [17, 20]. Furthermore, recreational football involving high-intensity bouts of exercise interspersed with activities performed at lower intensity for recovery, may constitute a viable training activity for improving HR_Rec_ [18, 21].

To the best of these study authors’ knowledge, no research has been published with the aim of evaluating the validity and sensitivity (i.e. external responsiveness) of HR_Rec_ in recreational football players. Information about the validity, sensitivity and applicability of HR_Rec_ monitoring in recreational football would be of great practical importance for the control, regulation and implementation of successful training programmes.

The main aim of this study was therefore to examine the association between HR_Rec_ values obtained at arbitrarily chosen time points after intermittent maximal field tests with VO_2max_ in adult recreational football players (convergent construct validity) in the untrained and trained states (i.e. longitudinal construct validity). Recovery HR was assessed using field tests deemed to induce exhaustion and popularly used in recreational football interventions (i.e. Yo-Yo intermittent tests) [17, 20]. An effect of individual and training-induced cardiorespiratory fitness improvements on HR_Rec_ was assumed as working hypothesis [13].

## Methods

### Participants

In this study, thirty-two male adults (age 40 ± 6 years, VO_2max_ 41.74 ± 5.72 ml·kg^-1^·min^-1^, body mass 82.7 ± 9.8 kg, stature 173.3 ± 7.4 cm, systolic and diastolic blood pressure 125 ± 11 and 74 ± 8 mmHg, respectively) volunteered to participate. The participants were tested at the untrained and trained states, i.e., before and after engaging in a 12-week recreational football training-based intervention. The untrained state (baseline conditions, i.e., pre-intervention) was defined as the participants having less than 20 min of exercise on 3 or more days a week [22]. All the participants were familiarised with the procedures used in the investigation during the two weeks before the commencement of the study by performing submaximal versions of the treadmill test and the Yo-Yo intermittent tests. The participants gave their written informed consent to participate in the study, which was conducted in accordance with the Declaration of Helsinki, and ethical approval was provided by the Ethics Committee of the Faculty of Sport, University of Porto (Porto, Portugal). All participants were informed of the risks and benefits of participating and made aware that they could withdraw from the study at any time without penalty.

### Design

In this study, HR_Rec_ was determined as the difference between the Yo-Yo intermittent tests’ peak HR (HR_peak_) or maximal HR (HR_max_), depending on whether HR_max_ or only HR_peak_ was reached during the test conditions, and post-exhaustion HR at selected time points, i.e. 30s (HR30), 60s (HR60) and 120s (HR120) after the end of the tests [3, 9]. Specifically, HR_Rec_ (i.e. ΔHR_Rec_ = peak/maximal HR minus post-exhaustion HR value) was reported in absolute values (beats·min^-1^) and as a percentage of HR_peak_ or HR_max_ (%HR_Rec_) reached during the tests [23]. With the aim of evaluating the proposed levels of validity, data normalisation was performed using HR_peak_ and HR_max_ in either the untrained or trained states. Maximal HR (HR_max_) was assessed as the maximal value reached across the testing conditions (i.e. Yo-Yo intermittent tests or the treadmill test for VO_2max_ assessment), using a multiple approach, as suggested by Póvoas et al. [16], in recreational football. HR_peak_ refers to the maximal value reached during a testing condition that requires maximal effort, but that is below the maximal reached by the participant in all testing conditions.

The magnitude of HR_60_ was rated for clinical importance using the cut-off values suggested by Cole et al. [3, 24]. Given the relatively active recovery observed post-exhaustion during the field tests (deceleration and spontaneous ambulation), abnormality was considered when HR_60_ was ≤12 beats·min^-1^ [3, 8].

The intensity and duration of the exercise used to induce HR_Rec_ has been considered as a confounding variable [13]. With the aim of examining the interest in using intermittent endurance field tests in assessing HR_Rec_, three intermittent versions of the Yo-Yo test were considered [17], namely levels 1 and 2 of the Yo-Yo intermittent endurance test (YYIE1 and YYIE2, respectively) and the Yo-Yo intermittent recovery test level 1 (YYIR1). The field test protocols were assumed to induce similar aerobic demands with different anaerobic involvement and time to exhaustion in order to stress different HR_Rec_ [13, 17].

After the baseline (i.e. untrained status) VO_2max_ and field testing, the participants engaged in a recreational football training intervention (2‒3 60-min weekly sessions) and were retested after 12 weeks of training to access the responsiveness of the selected variables (i.e., pre- and post-intervention). The training intervention was carried out according to the guidelines suggested by Krustrup et al. [18, 19, 25] for recreational football interventions with male participants.

### Testing procedures

The field tests (Yo-Yo intermittent tests) and the treadmill test for VO_2max_ (TT) assessment were performed in random order with at least 4 days (i.e., 4–6 days) of recovery in between. Test standardisation was achieved by performing the Yo-Yo intermittent tests on the same artificial football pitch and at the same time of day for circadian performance consistency. Furthermore, a standardised warm-up consisting of 10 min of running at different intensities and with changes of direction preceded each Yo-Yo intermittent test. Two minutes of passive rest were considered for each of the participants, before the start of the field tests. On the day before testing, the players refrained from vigorous physical activity.

The proposed Yo-Yo intermittent tests differ in their initial running speed and progression, and the between-bouts (40 m) recovery lasts 5–10 s, during which the participants are asked to cover 5–10 m. The Yo-Yo intermittent test protocols were implemented according to the procedures suggested by Krustrup et al. [26–28].

The TT (HP Cosmos Quasar, Nussdorf, Germany) consisted of 3 min of walking at 5 km·h^-1^ and 2 min of running at 8 km·h^-1^ with 0% inclination, and then alternating between increases in speed (1 km·h^-1^) and inclination (1%) every 30 s until voluntary exhaustion. Expired respiratory gas fractions were measured using an open-circuit breath-by-breath automated gas analysis system (Quark CPET, Cosmed, Rome, Italy). Attainment of VO_2max_ was assumed when the participants achieved a plateau in VO_2_ despite an increase in exercise intensity and at least one of the following criteria: a respiratory exchange ratio (RER) greater than 1.10 and RPE equal to or higher than 7 [29, 30]. The highest 15-s VO_2_ during the final stages of the test was considered as proof of individual VO_2max_ [16, 17]. Data analysis was performed with manual inspection of each TT data file using an Excel file (Microsoft, Redmont, USA).

Attainment of individual maximal effort during field tests was considered when the participants, at their subjective exhaustion, reported a rating of perceived exertion (RPE) equal to or higher than 7, at a 0–10 scale or had a HR_peak_ equal to or higher than 90% of their age-predicted HR_max_. Visual inspection of HR profile was performed to assess possible artefacts and to evaluate possible HR plateau and peak.

All exercise HRs were recorded at 1-s intervals using Polar Team System 2 HR monitors (Polar Electro Oy, Kempele, Finland). The players were allowed to drink water ad libitum in order to ensure proper hydration under all the exercise conditions considered in this study. After the completion of the field tests participants were instructed and guided to stay with minimal movement to standardise the recovery (2−3 min). No drinking was allowed during the recovery period.

### Training intervention

After baseline testing (i.e. laboratory and field testing, *n* = 32), the participants engaged in a recreational football intervention comprising 2–3 60-min training sessions per week in the form of 45-min small-sided games played on an artificial pitch (7v7; 43 x 27 m pitch, 83 m^2^ per player) [31]. The training intervention was conducted over 12 weeks, and the intensity of the sessions was monitored using HR monitors and the subjective internal load estimated by the RPE method [32]. All participants repeated all the test procedures post-intervention in the week after the completion of the last recreational football training session. Participants were advised to follow the guidelines followed at baseline testing.

### Statistical analyses

Results are expressed as means±standard deviations (±SD) and 95% confidence intervals (95% CI). Normality assumption was verified using the Shapiro-Wilk W-test. A repeated-measurements analysis of variance (ANOVA) with post-hoc Bonferroni test was used to compare HR_Rec_ across the tests’ recovery time points (i.e. HR_30,_ HR_60,_ HR_120_). Practical differences were assessed as partial eta squared (η^2^_p_) and magnitudes rated as follows: η^2^_p_≥0.14 large effect, 0.14>η^2^_p_≥0.06 medium effect, 0.06>η^2^_p_≥0.01 small effect and η^2^_p_<0.01 trivial effect [33]. Pearson correlation (r) was used to assess the associations between variables. The magnitude of the reported effects was described using the Hopkins et al. [34] criteria. Within-test conditions variability was expressed as coefficient of variation (%CV). Relative reliability was assessed using the intraclass correlation coefficient (ICC_3,1_) with 95% CI [35, 36]. According to Landis and Kock [37], ICC values of 0.00–0.20, 0.21–0.40, 0.41–0.60, 0.61–0.80, 0.81–1.00 were considered as slight, fair, moderate, substantial and almost perfect, respectively. The Cohen’s *d* was used to evaluate the effect size, with values above 0.8, between 0.8 and 0.5, between 0.5 and 0.2, and lower than 0.2 considered as large, moderate, small and trivial, respectively [24]. The smallest worthwhile change (SWC) in measurement was considered to test the practical difference between variables and calculated as 0.2 times the variable standard deviation [34]. Sample size estimation was performed for a sample power of 85% with an effect size of 0.50 at a significance level of 5%, resulting in 29 participants to be recruited. Significance was set at 5% (P< 0.05).

## Results

The participants showed a relative mean attendance of 73 ± 15% (26 ± 5 total training sessions out off a maximum of 36) with a weekly average of 2.2 ± 0.5 training sessions. The VO_2max_ (8.6%, ~1 MET) and Yo-Yo tests performances were significantly (large) improved (23, 37 and 35% for YYIE1, YYIE2 and YYIR1, respectively) after the training intervention (Table 1). A large and significant decrement (-2%) in HR_max_ was detected after 12 weeks of recreational football training (Table 1). Yo-Yo test HR_peak_ was significantly decreased (3, 1 and 1% for YYIE1, YYIE2 and YYIR1, respectively) post-intervention (small to large). The relative reliability (pre- to post-intervention) of the above variables was substantial to almost perfect (ICC>0.71).

**Table 1:** Pre- to post-training intervention changes in the considered variables.

Variable	Pre	Post	Diff	95%CI Diff	P value	*d*	ICC	TEM
**VO**_**2max**_ (ml·kg^-1^·min^-1^)	41.7±5.7	45.3±5.8	3.6±3.8	(-4.9; -2.3)	<0.0001	1.0	0.81 (0.64–0.90)	6.1 (4.9–8.2)
**HR**_**max**_ (beats·min^-1^)	186±10	182±10	4.1±3.9	(2.7–5.5)	<0.0001	1.0	0.93 (0.86–0.96)	1.5 (1.2–2.0)
**YYE1-HR**_**peak**_ (beats·min^-1^)	184±10	180±10	4.7±5.1	(2.8–6.5)	<0.0001	0.8	0.88 (0.77–0.94)	2.0 (1.6–2.7)
**YYE2-HR**_**peak**_ (beats·min^-1^)	181±11	179±10	2.3±4.8	(0.6–4.0)	0.01	0.4	0.90 (0.80–0.95)	1.9 (1.5–2.5)
**YYR1-HR**_**peak**_ (beats·min^-1^)	179±11	176±12	2.3±5.9	(0.2–4.5)	0.03	0.6	0.87 (0.74–0.93)	2.4 (1.9–3.2)
**YYE1-HR**_**30**_ (beats·min^-1^)	168±13	167±13	1.4±9.1	(-1.9–4.6)	0.41	0.1	0.75 (0.55–0.87)	3.9 (3.1–5.2)
**YYE1-HR**_**60**_ (beats·min^-1^)	150±16	147±16	3.2±11.6	(-1.0–7.4)	0.13	0.4	0.74 (0.54–0.87)	5.6 (4.5–7.5)
**YYE1-HR**_**120**_ (beats·min^-1^)	132±17	121±15	10.5±13.3	(5.7–15.3)	0.0001	0.8	0.67 (0.42–0.82)	7.4 (5.9–10)
**YYE2-HR**_**30**_ (beats·min^-1^)	170±13	165±10	4.3±7.9	(1.5–7.1)	0.004	0.7	0.77 (0.58–0.88)	3.3 (2.7–4.4)
**YYE2-HR**_**60**_ (beats·min^-1^)	156±17	149±14	7.2±10.3	(3.5–10.9)	0.0004	0.7	0.79 (0.61–0.89)	5.2 (4.2–7.0)
**YYE2-HR**_**120**_ (beats·min^-1^)	134±22	126±16	8.0±13.8	(3.0–13.0)	0.003	1.1	0.75 (0.55–0.87)	9.0 (7.2–12.2)
**YYR1-HR**_**30**_ (beats·min^-1^)	162±13	163±14	0.6±9.3	(-2.7–4.0)	0.71	0.1	0.77 (0.58–0.88)	4.2 (3.3–5.6)
**YYR1-HR**_**60**_ (beats·min^-1^)	147±16	145±17	1.4±11.2	(-2.6–5.4)	0.48	0.2	0.77 (0.58–0.88)	5.7 (4.6–7.7)
**YYR1-HR**_**120**_ (beats·min^-1^)	125±16	125±13	0.2±11.4	(-3.9–4.3)	0.91	0.0	0.70 (0.47–0.84)	6.7 (5.4–9.1)
**ΔHR**_**peak**_ (beats·min^-1^)								
**YYE1-HR**_**30**_	16±5	13±5	3.4±6	(1.1–5.6)	0.004	0.5	0.33 (-0.02–0.61)	42.4 (32.7–59.9)
**YYE1-HR**_**60**_	34±10	32±10	1.5±9	(-1.7; 4.7)	0.34	0.2	0.61 (0.33–0.79)	1.1 (0.9–1.5)
**YYE1-HR**_**120**_	49±14	60±12	-11±13	(-15.0; -5.7)	<0.0001	0.8	0.48 (0.17–0.71)	21.4 (16.8–29.3)
**YYE2-HR**_**30**_	12±5	14±4	-2±6	(-4.3–0.3)	0.08	0.33	0.08 (-0.27–0.42)	47.7 (36.7–68.0)
**YYE2-HR**_**60**_	25±9	30±8	-5±8	(-7.7; -2.1)	0.001	0.65	0.60 (0.32–0.78)	23.7 (18.6–32.7)
**YYE2-HR**_**120**_	47±15	55±11	-8±14	(-13.0; -3.0)	0.003	0.60	0.46 (0.14–0.69)	24.1 (18.9–33.3)
**YYR1-HR**_**30**_	16±6	13±5	3±7	(0.5–5.5)	0.02	0.42	0.17 (-0.19–0.48)	45.7 (35.2–64.9)
**YYR1-HR**_**60**_	32±9	31±10	1±9	(-2.2–3.9)	0.58	0.12	0.62 (0.35–0.79)	0.9 (0.7–1.2)
**YYR1-HR**_**120**_	56±10	57±11	-1±10	(-4.9–2.3)	0.46	0.10	0.57 (0.29–0.77)	14.8 (11.7–20.1)
**ΔHR**_**max**_(beats·min^-1^)								
**YYE1-HR**_**30**_	18±7	15±7	3±8	(-0.1–5.6)	0.05	0.39	0.42 (0.08–0.66)	45.4 (35.0–64.5)
**YYE1-HR**_**60**_	36±11	35±11	1±11	(-2.9–4.8)	0.62	0.09	0.52 (0.21–0.73)	30.1 (23.5–41.9)
**YYE1-HR**_**120**_	54±13	61±12	-6±13	(-10.9; -1.8)	0.008	0.55	0.48 (0.17–0.71)	18.3 (14.4–25.0)
**YYE2-HR**_**30**_	16±6	17±5	-0.1±8	(-2.9–2.6)	0.92	0.13	0.05 (-0.30–0.39)	48.3 (37.2–68.9)
**YYE2-HR**_**60**_	30±11	33±8	-3±10	(-6.6–0.5)	0.09	0.32	0.49 (0.18–0.72)	27.0 (21.1–37.4)
**YYE2-HR**_**120**_	52±16	55±16	-3±14	(-8.0–2.0)	0.23	0.22	0.63 (0.37–0.80)	22.6 (17.8–31.2)
**YYR1-HR**_**30**_	24±8	19±6	5±10	(1.2–8.3)	0.01	0.51	0.02 (-0.33–0.36)	43.1 (33.3–61.0)
**YYR1-HR**_**60**_	39±10	37±11	3±12	(-1.4–6.9)	0.19	0.17	0.39 (0.05–0.65)	25.7 (20.1–35.5)
**YYR1-HR**_**120**_	61±10	57±11	4±11	(-0.2–8.0)	0.06	0.35	0.43 (0.10–0.67)	16.6 (13.1–22.6)
**YYIE1** (m)	1600±621	1969±757	369±321	(-485; -253)	<0.0001	1.2	0.90 (0.80–0.95)	14 (11.1–19.0)
**YYIE2** (m)	471±200	648±230	176±143	(-228; -125)	<0.0001	1.3	0.79 (0.62–0.89)	17 (13.7–23.7)
**YYIR1** (m)	674±298	909±374	235±267	(-331; -139)	<0.0001	0.9	0.71 (0.47–0.84)	21 (16.5–28.8)

**VO**_**2max**_ **=** Maximal Oxygen Uptake; **HR**_**max**_ = Maximal Heart Rate; **YYIE1 =** Yo-Yo intermittent Endurance Test Level 1; **YYIE2** = Yo-Yo intermittent Endurance Test Level 2; **YYIR1 =** Yo-Yo intermittent Recovery Test Level 1; **Diff =** Difference in absolute value; **95%CI** = 95% Confidence Interval of difference; ***d* =** Cohen *d*; **ICC** = Intraclass Correlation Coefficient; **TEM** = Typical Error of the Measurement as % Coefficient of Variation.

HR values during the considered recovery time points are reported in Table 2 as absolute (beats· min^-1^) and relative (% of tests’ HR_peak_ or HR_max_ values). Large and significant (p<0.0001) differences were reported for the test conditions across the selected recovery time points and on the two testing occasions (i.e. pre- and post-training intervention). Participants achieved 87–94, 79–86 and 67–74% of their HR_max_ or HR_peak_ values at HR_30_, HR_60_ and HR_120_, respectively. The corresponding absolute HR difference ranges were 11–24, 25–39 and 51–61 beats·min^-1^ for HR_30_, HR_60_ and HR_120_, respectively.

**Table 2:** Heart rate (HR) recovery values at selected recovery time points after the field tests and between their values differences pre- to post-training intervention. The HR values are reported as % of test peak HR (HR_peak_) and individual maximal HR (HR_max_).

Training status	Yo-Yo test	Variable	HR (beats·min^-1^)	%HR_peak_	%HR_max_	95%CI Diff	ΔHR_peak_	ΔHR_max_	η^2^_p_
**Pre-intervention**	**YYIE1**	HR_max_	186±10			(14–21)*^#^			0.86
		HR_peak_	184±10						
		HR_30_	168±13	91±3	90±4	(14–19)*	16	18	0.91
		HR_60_	151±16	80±10	81±6	(15–21)*	33	35	0.88
		HR_120_	132±17	71±7	71±7	(15–22)*	52	54	0.87
	**YYIE2**	HR_max_	186±10			(13–20)*^#^			0.88
		HR_peak_	181±11						
		HR_30_	170±13	94±3	91±3	(15–21)*	11	16	0.86
		HR_60_	156±17	86±6	84±6	(10–17)*	25	30	0.80
		HR_120_	134±22	74±10	72±8	(17–27)*	47	52	0.84
	**YYIR1**	HR_max_	186±10			(20–28)*^#^			0.91
		HR_peak_	179±11						
		HR_30_	162±13	91±3	87±4	(14–19)*	17	24	0.90
		HR_60_	147±16	82±5	79±6	(12–19)*	32	39	0.86
		HR_120_	125±16	70±6	67±6	(19–25)*	54	61	0.93
**Post-intervention**	**YYIE1**	HR_max_	182±10			(11–19)*^#^			0.82
		HR_peak_	180±10						
		HR_30_	167±13	93±3	92±4	(10–16)*	13	15	0.86
		HR_60_	147±16	82±6	81±6	(16–23)*	33	35	0.89
		HR_120_	121±15	67±6	67±7	(21–31)*	59	61	0.88
	**YYIE2**	HR_max_	182±10			(14–19)*^#^			0.92
		HR_peak_	179±10						
		HR_30_	165±10	92±3	91±3	(12–16)*	14	17	0.91
		HR_60_	149±14	83±5	82±5	(13–20)*	30	33	0.87
		HR_120_	126±16	71±7	69±7	(19–26)*	53	56	0.91
	**YYIR1**	HR_max_	182±10			(16–22)*^#^			0.90
		HR_peak_	176±12						
		HR_30_	163±14	92±3	89±4	(11–16)*	13	19	0.87
		HR_60_	145±17	82±6	80±7	(14–21)*	31	37	0.88
		HR_120_	125±13	71±6	69±6	(16–25)*	51	57	0.84

**HR** = Heart Rate; **HR**_**peak**_ = highest HR achieved by participants during the Yo-Yo tests; %**HR**_**peak**_ = Percentage of **HR**_**peak**_ achieved by participants at the set recovery time; **HR**_**30**_ = HR 30s after the end of the Yo-Yo test; **HR**_**60**_ = HR 60s after the end of the Yo-Yo test; **HR**_**120**_ = HR 120s after the end of the Yo-Yo test; **95%CI Diff** = 95% Confidence Interval of difference; * = p<0.0001 for the selected contrasts (HR_peak_ vs HR_30_, HR_30_ vs HR_60_, HR_60_ vs HR_120_); *^#^ = difference between HR_max_ and HR_30_ value at p<0.0001; **ΔHR**_**peak**_ = Difference between HR_peak_ and HR at the considered recovery time points in beats·min^-1^; **ΔHR**_**max**_ = Difference between HR_max_ and HR at the considered recovery time points in beats·min^-1^; **η**^**2**^_**p**_ = Partial Eta Squared.

Significantly higher (small) relative (%) post-training HR_30_ values were found in YYIE1 when using HR_peak_ or HR_max_ for normalising HR_Rec_ (Table 3). Higher (small, p<0.04) post-intervention %HR_30_ values in YYIR1 were reported for both HR_peak_ and HR_max_. Lower and significant (p<0.04) post-intervention %HR_60_ values were seen for HR_peak_ (moderate) and HR_max_ (small) in the YYIE2 test. When considering %HR_120_, a significant and moderate decrement was evident in YYIE1 for both HR_peak_ and HR_max_. The YYIE2%HR_120_ was small and significantly lower when considering HR_peak_ for normalisation.

**Table 3:** Heart rate (HR) recovery values (%) at selected time points before (pre) and after (post) the training intervention and considering test peak HR (HR_peak_) and across tests maximal HR (HR_max_), as normalizing variables.

Normalizing variable	HR_Rec_	Pre (beats·min^-1^)	Post (beats·min^-1^)	95%CI Diff	P value	*d*
**HR**_**peak**_	**YYIE1-HR**_**30**_	91±3	93±3	0.44–2.82	0.01	0.49
	**YYIE2-HR**_**30**_	94±3	92±2	-2.40–0.15	0.08	0.51
	**YYIR1-HR**_**30**_	91±3	92±3	0.17–3.07	0.03	0.40
**HR**_**max**_	**YYIE1-HR**_**30**_	90±4	92±4	-2.85–0.17	0.08	0.32
	**YYIE2-HR**_**30**_	91±3	91±3	-1.48–1.55	0.97	0.01
	**YYIR1-HR**_**30**_	87±4	89±4	0.38–4.12	0.02	0.43
**HR**_**peak**_	**YYIE1-HR**_**60**_	80±10	82±6	-1.40–5.21	0.25	0.22
	**YYIE2-HR**_**60**_	86±6	83±5	1.30–4.60	0.001	0.64
	**YYIR1-HR**_**60**_	82±5	82±6	-1.52–2.07	0.75	0.06
**HR**_**max**_	**YYIE1-HR**_**60**_	81±6	81±6	-2.03–2.09	0.98	0.00
	**YYIE2-HR**_**60**_	84±6	82±5	0.20–4.05	0.03	0.41
	**YYIR1-HR**_**60**_	79±6	80±7	-1.38–3.13	0.43	0.14
**HR**_**peak**_	**YYIE1-HR**_**120**_	71±7	67±6	-6.13;-1.74	0.01	0.65
	**YYIE2-HR**_**120**_	74±10	71±7	-5.89;-0.69	0.02	0.49
	**YYIR1-HR**_**120**_	70±6	71±6	-1.23–3.16	0.37	0.16
**HR**_**max**_	**YYIE1-HR**_**120**_	71±7	67±7	-6.57;-1.67	0.002	0.61
	**YYIE2-HR**_**120**_	72±10	69±7	-0.01–5.32	0.05	0.39
	**YYIR1-HR**_**120**_	67±6	69±6	-0.78–3.84	0.19	0.24

**HR** = Heart Rate; **HR**_**Rec**_ = recovery HR; **HR**_**peak**_ = highest HR achieved by participants during the Yo-Yo test; **HR**_**max**_ = highest HR achieved by participants during the Yo-Yo tests and Treadmill test; **HR**_**30**_ = HR 30s after the end of the Yo-Yo test; **HR**_**60**_ = HR 60s after the end of the Yo-Yo test; **HR**_**120**_ = HR 120s after the end of the Yo-Yo test; **95%CI Diff** = 95% Confidence Interval of difference; ***d*** = Cohen’s *d*.

At baseline, VO_2max_ was not significantly associated (trivial to moderate) with HR_Rec_ at the selected time points, expressed as test HR_peak_ or HR_max_, in any of the considered field-testing conditions (i.e. YYIE1, YYIE2 and YYIR1, Table 4). The lack of significant (p>0.05) correlations persisted in the trained state.

**Table 4:** Associations between maximal oxygen uptake (VO_2max_) and heart rate (HR) recovery at different time points (i.e., 30, 60 and 120 seconds) after the field tests using the test HR peak (HR_peak_) and maximal HR (HR_max_) as normalising variables in the untrained (i.e., pre-intervention) and trained state (i.e., post-intervention).

		HR recovery								
Normalising Variables	VO_2max_	YYIE1_30_	YYIE1_60_	YYIE1_120_	YYIE2_30_	YYIE2_60_	YYIE2_120_	YYIR1_30_	YYIR1_60_	YYIR1_120_
**HR**_**peak**_										
	**Pre**	0.05	-0.02	-0.04	0.33	0.30	0.31	-0.05	0.20	0.05
	***95%CI***	-0.30–0.39	-0.37–0.33	-0.38–0.31	-0.02–0.61	-0.05–0.59	-0.04–0.59	-0.39–0.31	-0.16–0.52	-0.30–0.39
	***P value***	0.79	0.91	0.83	0.07	0.091	0.084	0.79	0.28	0.79
	**Post**									
	***95%CI***	-0.19–0.49	-0.13–0.53	-0.28–0.41	-0.49–0.18	-0.23–0.46	-0.12–0.54	-0.05–0.59	-0.16–0.52	-0.29–0.41
	***P value***	0.36	0.22	0.69	0.34	0.46	0.18	0.10	0.27	0.71
**HR**_**max**_										
	**Pre**	0.11	0.05	-0.01	0.16	0.22	0.27	0.01	0.20	0.07
	***95%CI***	-0.25–0.44	-0.30–0.39	-0.35–0.34	-0.20–0.49	-0.14–0.53	-0.09–0.57	-0.34–0.36	-0.16–0.52	-0.28–0.41
	***P value***	0.55	0.77	0.97	0.37	0.22	0.13	0.95	0.27	0.69
	**Post**	0.19	0.24	0.09	0.14	-0.17	0.24	0.17	0.15	0.03
	***95%CI***	-0.17–0.50	-0.12–0.54	-0.27–0.42	-0.22–0.46	-0.49–0.19	-0.12–0.54	-0.19–0.49	-0.21–0.48	-0.32–0.38
	***P value***	0.31	0.19	0.63	0.46	0.34	0.18	0.35	0.40	0.85

**HR** = Heart Rate; **HR**_**peak**_ = highest HR achieved by participants during the Yo-Yo tests; **HR**_**max**_ = highest HR achieved by participants across the considered tests; **YYIE1** = Yo-Yo intermittent endurance test level 1; **YYIE2** = Yo-Yo intermittent endurance test level 2; **YYIR1** = Yo-Yo intermittent recovery test Level 1; **95%CI Diff** = 95% confidence interval.

Using the ≤12 beats·min^-1^ criterion to qualitatively evaluate HR_Rec_, only 3‒6% and 3% of the participants reported the supposed abnormalities in HR_60_ during the pre-intervention YYIE2 and post-intervention YYIE1, respectively (Table 5).

**Table 5:** Frequency (count and %) of participants with heart rate (HR) recovery difference ≤12 beats·min^-1^ in the untrained and trained state (i.e., at pre- and post-intervention).

		Pre			Post		
Variable		HR_30_	HR_60_	HR_120_	HR_30_	HR_60_	HR_120_
**YYIE1**	HR_peak_	9 (28%)	0	0	19 (59%)	1 (3%)	0
	HR_max_	7 (22%)	0	0	17 (53%)	1 (3%)	0
**YYIE2**	HR_peak_	22 (69%)	2 (6%)	0	12 (38%)	0	0
	HR_max_	9 (28%)	1 (3%)	0	8 (25%)	0	0
**YYIR1**	HR_peak_	7 (22%)	0	0	15 (47%)	0	0
** **	HR_max_	3 (9%)	0	0	5 (16%)	0	0

**HR** = Heart Rate; **HR**_**peak**_ = highest HR achieved by participants during the Yo-Yo tests; **HR**_**max**_ = highest HR achieved by participants across the considered tests; **YYIE1** = Yo-Yo intermittent endurance test level 1; **YYIE2** = Yo-Yo intermittent endurance test level 2; **YYIR1** = Yo-Yo intermittent recovery test Level 1; **HR**_**30**_ = HR 30s after the end of the Yo-Yo test; **HR**_**60**_ = HR 60s after the end of the Yo-Yo test; **HR**_**120**_ = HR 120s after the end of the Yo-Yo test.

## Discussion

This is the first study to examine the validity and sensitivity of using intermittent endurance field tests’ post-exhaustion HR_Rec_ values to characterise cardiorespiratory fitness in male participants that volunteered for a recreational football intervention, in the trained and untrained states [13, 17]. These tests were supposed to induce similar maximal aerobic demands and different anaerobic loads [38, 39]. The main finding was that no associations of significance or practical importance were found between HR_Rec_ and cardiorespiratory fitness in either the untrained or trained states. The reported significant, mainly moderate to large, changes in post-intervention HR_peak_ and HR_max_ affected the representation of HR_Rec_ values magnitude, suggesting a multifaceted interpretation of HR_Rec_. The occurrence of HR_Rec_ abnormalities (i.e. ≤12 beats·min^-1^ for HR_60_) was dependent on the field test performed and training state (i.e. untrained or trained). This suggests that caution is advised when considering fixed recovery HR count as a reference for characterising poor HR_Rec_ in healthy male recreational football players in the age span here considered (30–50 years).

In this study, the relative reliability of VO_2max_, HR_max_ and Yo-Yo HR_peak_ pre- to post-intervention was almost perfect (ICC, 0.81–0.93), supporting the consistency of participants’ training-induced changes ranking [37]. Interestingly, significant decrements in HR_max_ (large) and Yo-Yo HR_peak_ (small-to-large) were found at post-intervention (Table 1) [40]. These results provide evidence of the internal validity of this study design and confirm the applicability of the Yo-Yo intermittent tests in recreational football [16, 17, 20].

Bosquet et al. [23] assessed HR_Rec_ reliability by replicating a maximal treadmill test at least 72 hours apart in healthy subjects. A general low relative reliability of ΔHR_Rec_ variables was reported, except when considering absolute HR_Rec_ values (beats·min^-1^), with ICC ranging between 0.68 and 0.83 [23]. In line with the cited study, we found a substantial agreement between pre-to-post absolute HR_Rec_ values (0.67–0.79) across the post-exhaustion time points [23]. Similarly, mainly slight to moderate agreement (0.17–0.61 and 0.05–0.63 using HR_peak_ and HR_max_, respectively) was reported for ΔHR_Rec_ across the Yo-Yo tests and recovery time points. These results (i.e. long-term relative reliability) add evidence of the poor reliability of ΔHR_Rec_ values when used to characterise HR_Rec_ after maximal testing [23]. This finding is of practical relevance as, in the clinical set-up, HR_Rec_ efficiency is mainly reported as ΔHR_Rec_ [3, 14, 41].

HR_Rec_ is deemed to be affected by the fitness level and training status of subjects of different age, gender and health condition [5, 7, 13, 42]. Cross-sectional studies found cardiorespiratory fitness as a possible cause for the faster HR_Rec_ in athletic populations compared to untrained or inactive subjects [13]. Darr et al. [41] reported an effect of VO_2max_ level on HR_Rec_ with trained subjects (>60 ml·kg^-1^·min^-1^ mean VO_2peak_), reporting a faster decrement in HR than in untrained subjects. Interestingly, the untrained male subjects’ mean VO_2max_ values (40 ml·kg^-1^·min^-1^) were similar to those of this study’s recreational football participants at pre-intervention evaluations (i.e. untrained status), suggesting an effect of training status on HR_Rec_.

This study’s findings did not provide empirical evidence of an association between cardiorespiratory fitness improvements and HR_Rec_ variations in recreational football players. This was supported by the analyses performed in both the untrained and trained status and occurs irrespective of consideration for physiological (i.e. VO_2max_) or performance (Yo-Yo tests) changes. Additionally, this study’s results are in line with those of Hautala et al. [43] in inactive subjects who trained for 2 weeks on an intensive endurance programme and reported no variations in HR_60_, despite significant positive VO_2max_ changes (+8%). Similarly, 3 weeks of intensive football training did not provide any changes in HR_Rec_ in competitive young football players [44]. Again, comparison with other studies addressing HR_Rec_ may be confounding, as different research designs and HR_Rec_ assessment tests were used [45].

The reported large and significant reduction in HR_max_ and HR_peak_ across the Yo-Yo tests further supports the previously reported findings of training-induced changes in heart physiology [40]. Although not based on robust mechanistic evidence, the reported significant and practical important decrement in HR_max_ could have been the result of variations in short-term neurological changes related to variation in parasympathetic and sympathetic nervous systems interplay [40]. Short-term effects of recreational football on players’ heart anatomy and physiology, may have also played a role [46]. However, this recreational football intervention revealed a limited effect on HR_Rec_, with moderate to large changes in absolute HR_Rec_ values observed only for HR_120_ after YYIE2 and YYIE1 and for HR_30_ and HR_60_ after YYIE2 (Table 1), suggesting a test-dependent effect on HR_Rec_. This was further supported by ΔHR_Rec_ analyses, revealing significantly higher post-intervention values only for a few test conditions, i.e. HR_60_ in YYIE2 and HR_120_ in both YYIE2 and YYIE1, when considering HR_peak_ as a reference for normalisation. Interestingly, a faster post-training intervention HR_Rec_ was only evident for the YYIE1 ΔHR_120_ when considering HR_max_. The variations in HR_Rec_ across the tests and testing time points were also evident when considering %HR_Rec_ values with a general increase (trivial to moderate) in post-intervention values.

Improvements in VO_2max_ and aerobic performance as a consequence of endurance training and recreational football practice were shown to be associated with significant decrements in HR_max_ and changes in HR_peak_ in field tests [16, 20, 40]. In this study, large and significant decrements in HR_max_ were detected, providing practical relevance of changes in the range of 3‒6 beats·min^-1^ (SWC 2 beats·min^-1^). Given the period of the considered change (12 weeks), a reassessment of HR_max_ every 12 weeks may be advisable for controlling and regulating exercise intensity in recreational football interventions. The reported changes in HR_max_ were paralleled by moderate to large decrements (p<0.04) in Yo-Yo HR_peak_ post-intervention, further suggesting caution when choosing ΔHR_Rec_ to profile HR_Rec_. Indeed, variations in peak HR values as an effect of training, alongside with the reported fair-to-moderate HR_Rec_ relative reliability, discourage consideration of HR_Rec_ as raw data difference values [23].

Cole et al. [3, 8] provided longitudinal evidence of an association between decrements in HR_Rec_ and reductions in all-cause and cardiovascular mortality. Furthermore, the same authors demonstrated the predictive strength of absolute HR cut-off values when considering HR_60_ and HR_120_. Using the suggested cut-off values (i.e. ≤12 beats·min^-1^), we only found 3–6% of abnormalities in HR_60_ across the field tests. The training intervention and the associated increase in VO_2max_ (~9%) and corresponding decrement in HR_max_ (~3%) produced a remarkable reduction in the initial HR_60_ abnormalities, when considering a highly demanding test like YYIE2 (Table 5). Indeed, no HR_60_ abnormalities were detected in the participants when examining pre-intervention HR_60_ after the YYIE1 and YYIR1 tests. Interestingly, only 3% of participants reported HR_60_ abnormalities in YYIE1 HR_60_ post-intervention, suggesting a sort of independence between cardiorespiratory fitness improvement and HR_60_ abnormalities. The resulting occurrence of HR_60_ abnormalities may have been the direct consequence of the participants’ health-related inclusion criteria. However, the lower prevalence of abnormal HR_60_ values at post-intervention might be considered as evidence of a possible positive effect of recreational football practice on reducing potential health-related risk factors. The reported test-related prevalence of HR_60_ abnormalities has practical importance for preventive medicine that warrants future studies [3, 8, 10].

However, the normative value proposed by these authors was derived from a population of male inactive subjects who were approximately 20 years older than the recreational football participants considered in the present study, which promotes the interest in population-specific normative values for tracking abnormalities in HR_Rec_ [3, 10].

The intermittent nature of recreational football, involving high-intensity bouts of exercise interspersed with activities performed at lower intensity for recovery, or less demanding match-related actions, potentially would be effective for improving HR_Rec_ [18]. However, this study results did not provide evidence for enhanced HR_Rec_ as result of a typical recreational football intervention. This was probably the consequence of considering the training-induced variations in HR_peak_ and HR_max_ when profiling the HR_Rec_ variables at post-intervention [40, 47]. Further studies with larger samples and a mechanistic design, are warranted to understand the variation in heart physiology provided by a casually intermittent activity such as recreational football in different populations. Additionally, the use of a control group would be useful in future studies to fully understand the nature of HR_Rec_ and related variables.

These findings question the use of HR_Rec_ as indicator of cardiorespiratory fitness level (see if you agree) or training-induced changes in healthy subjects participating in a recreational football intervention or in recreational players during the training process. The observed large changes (26–43%) in field test performance suggest sensitivity in tracking the aimed enhancement of the individual aerobic performance in recreational football training interventions. Changes in YYIE1 and YYIR1 performance were moderately and significantly associated with changes in HR_60_ (r = 0.36, P = 0.045) and HR_120_ (r = 0.38, P = 0.034)_,_ respectively. These results support the general picture of a limited association between changes in cardiorespiratory fitness level in recreational football players and HR_Rec_ variables.

In the published literature, HR_Rec_ assessment is reported as absolute values (beats·min^-1^) [3, 7, 13, 23]. Despite the practical interest of considering absolute values, differences in individual HR_max_ may provide biased data supposedly producing false positive results. Heart rate recovery normalisation using peak test HR or HR_max_ may potentially be the solution for avoiding biased data that may affect training prescription and clinical diagnosis. However, in this study the deliberate use of absolute or HR_max_ derived HR_Rec_ did not provide differences in the information supposed to have clinical importance. Inter-subject variability in test HR_peak_ (~6%) may have been the cause of the reported limited effect of data normalisation on the considered variables. The practical interest of the effect of reporting HR_Rec_ data deserves further studies with populations of different age and cardiorespiratory fitness level.

Attention should be paid when considering cut-off values (i.e. 12 or 43 beats·min^-1^) to qualitatively characterise the risk of developing cardiovascular diseases [3, 8–10]. A bias-limiting indicator of the cardiorespiratory risk could be developed using individual maximal values. Unfortunately, the reduced data variability of this study’s participants was not helpful in providing meaningful guidelines and further studies are warranted. However, the age range of this study’s participants (5.6 years, standard deviation range 22 years) may have affected the results, as in age-independent groups subjects with higher HR_max_ reported better absolute HR_Rec_. In this study, a moderate association was reported between HR_max_ and HR_Rec_.

Future training studies should also investigate the possibility to find more meaningful HR_Rec_ reference time points as suggested in a cross-sectional study by Ostojic et al [48]. This would be of specific interest, as short-term HR_Rec_ (i.e., as short as 20s) has been reported to be faster in athletes of intermittent sports (i.e., basketball, soccer and team handball) with at least four years of participation in these sports [48]. Given that, knowledge about the applicability of the short-term HR_Rec_ concept in previously untrained subjects participating in a training intervention, using exclusively intermittent exercise like recreational football, would be of great practical interest.

## Conclusions

It was not possible with this study design to support the validity of tracking post-exhaustion HR_Rec_ to estimate individual aerobic fitness (VO_2max_), either in the untrained or trained status (i.e. pre- and post-intervention, respectively). Indeed, no significant and practically important associations were found between HR_Rec_ variables and recreational football players’ VO_2max_. This study’s results are in line with those reported in the athletic populations, suggesting HR_Rec_ as a “per se” physiological adaptation that is independent of VO_2max_ level and changes [13].

Given the interest of this issue for public health, further studies involving a larger number of participants followed for a longer time are warranted. From the practical point of view, HR_Rec_ is a reliable variable in the short-term (i.e. 12 weeks), nevertheless, it is not associated with the improvement in aerobic fitness in this population of recreational football players.

Although this study’s results refer to recreational football players, the information obtained may be of interest for all professionals dealing with health-enhancing strategies evaluated under field conditions.

ΔHR_Rec_ is considered as the variable for tracking the efficiency of the physiological processes that underpin HR_Rec_. This study’s results suggest that caution is advised when considering ΔHR_Rec_, as this variable may be affected by the concomitant reductions in HR at exhaustion values and, consequently, bias the reported differences.
